# A predictive model for secondary RNA structure using graph theory and a neural network

**DOI:** 10.1186/1471-2105-11-S6-S21

**Published:** 2010-10-07

**Authors:** Denise R Koessler, Debra J Knisley, Jeff Knisley, Teresa Haynes

**Affiliations:** 1 Department of Mathematics and Statistics, East Tennessee State University, Johnson City, TN 37614, USA; 2 The Institute for Quantitative Biology, East Tennessee State University, Johnson City, TN 37614, USA

## Abstract

**Background:**

Determining the secondary structure of RNA from the primary structure is a challenging computational problem. A number of algorithms have been developed to predict the secondary structure from the primary structure. It is agreed that there is still room for improvement in each of these approaches. In this work we build a predictive model for secondary RNA structure using a graph-theoretic tree representation of secondary RNA structure. We model the bonding of two RNA secondary structures to form a larger secondary structure with a graph operation we call* merge.* We consider all combinatorial possibilities using all possible tree inputs, both those that are RNA-like in structure and those that are not. The resulting data from each tree merge operation is represented by a vector. We use these vectors as input values for a neural network and train the network to recognize a tree as RNA-like or not, based on the merge data vector. The network estimates the probability of a tree being RNA-like.

**Results:**

The network correctly assigned a high probability of RNA-likeness to trees previously identified as RNA-like and a low probability of RNA-likeness to those classified as not RNA-like. We then used the neural network to predict the RNA-likeness of the unclassified trees.

**Conclusions:**

There are a number of secondary RNA structure prediction algorithms available online. These programs are based on finding the secondary structure with the lowest total free energy. In this work, we create a predictive tool for secondary RNA structures using graph-theoretic values as input for a neural network. The use of a graph operation to theoretically describe the bonding of secondary RNA is novel and is an entirely different approach to the prediction of secondary RNA structures. Our method correctly predicted trees to be RNA-like or not RNA-like for all known cases. In addition, our results convey a measure of likelihood that a tree is RNA-like or not RNA-like. Given that the majority of secondary RNA folding algorithms return more than one possible outcome, our method provides a means of determining the best or most likely structures among all of the possible outcomes.

## Background

Our understanding of the role of RNA has changed and continues to be redefined. It was once believed that the sole purpose of RNA is to carry the information needed to construct a specific protein. This information is obtained from the protein’s gene and carried from the nucleus of the cell to the machinery outside the nucleus in the cell where the protein is then constructed. That is, RNA carried the protein’s code. It is now known that RNA is a major player in many gene regulatory networks and that there are numerous RNA structures whose function does not involve coding for a protein. Thus, we now refer to RNA as either coding or non-coding. Novel non-coding RNA structures are still being found and the number of non-coding RNA structures now exceeds the number of coding RNA [1]. The role of non-coding RNA in gene regulatory networks places the study of RNA in the forefront of efforts to understand the complexities in Systems Biology.

A number of algorithms have been developed to predict the secondary structure from the primary structure. Most of these algorithms use the thermodynamic parameters based on the principle that the most likely secondary structure is one having the minimal free energy. But some suggest the actual RNA secondary structure may have local instead of a global minimum free energy [2] and thus many algorithms try to simulate RNA folding processes by iteratively adding stems rather than pairings [3,4].

RNAalifold [5] integrates thermodynamic and phylogenetic information in a modified energetic model. There is general agreement that there is still room for improvement in each of these approaches [6]. The database RAG: RNA-As-Graphs represents secondary RNA using graph theory [7]. The details of the graph-theoretic representation are provided in the following section. We utilize the information in the RAG database to develop a novel predictive tool for secondary RNA structure. When our tool is applied to predict secondary RNA structures that are listed in the database, our results concur with the database. We then predict additional secondary RNA structures with our graph-theoretic based predictive tool. Given that most algorithms return several predicted secondary structures, ranking them in order of likelihood, we provide an additional tool to assist in the determination of which of the predicted structures is most likely.

### Modeling RNA

RNA structure is divided into three classes: primary, secondary and tertiary. The RNA sequence is the primary structure. The primary structure forms the secondary by folding back onto itself. When this folding occurs, it forms Watson-Crick base pairs with intervening unpaired regions. These regions occur in four types of structures known as hairpins, bulges, internal loops and junctions. Paired regions connecting these are usually referred to as stems. Secondary RNA structure can be represented by a two-dimensional drawing. Tertiary RNA, or the three dimensional RNA molecule, can be best described as being built from combinations of secondary structure. Thus, the study of the secondary structure of RNA has received and will continue to receive much attention.

Since secondary RNA is represented by a two-dimensional schematic, graph theory nicely lends itself as a modeling tool for secondary RNA structure. The basic skeletal structure of secondary RNA is captured by representing the stems as edges of the graph and the regions with unpaired bases as vertices. The resulting graph is a graph known as tree graph, or simply a tree. The RNA trees used in this work were first developed by Le et al. in [8] and Morosetti [9] to determine structural similarities in RNA.

To quantitatively organize and archive all possible RNA tree representations, it is necessary to first generate the collection of all possible trees for a given number of vertices (*n*). For example, for the set *n* = {2, 3, 4, 5, 6, 7, 8, 9}, there are a total of {1, 1, 2, 3, 6, 11, 23, 47} distinct trees, respectively [10]. The RAG database catalogs all tree structures for trees up to order 11 [7]. According to [7], the second eigenvalue λ_2_ measures a motif’s topological complexity. For example, a more linear tree graph has a lower λ_2_ value while a highly-branched tree graph has a high λ_2_ value. The RAG database catalogs each potential RNA motif by (*n*, λ_2_). For easy reference, each RNA motif has a specific index (*n*.*z*), where *z* represents an integer corresponding to the λ_2_ ranking.

The trees with 2 through 8 vertices have been fully classified as known (verified), candidate or non-candidate. The research compiled in [7] organizes all known, candidate and non-candidate RNA trees of order 8 or less by a color coding scheme. Red trees represent known RNA, blue trees are candidate RNA and black trees are non-candidate trees. A tree that is either a known tree or candidate tree is referred to as an RNA-like tree and a non-candidate tree is referred to as not-RNA-like. The remaining trees on 9 or higher vertices have not be grouped into these three categories. This catalog of RNA trees is intended as a tool for searching existing RNAs and to stimulate the search for candidate RNA motifs not yet discovered in nature or a laboratory.

There are a number secondary RNA structure prediction algorithms available online such as Zuckers MFold and Vienna RFold. Given the primary RNA sequence, the web server will return a list of predicted secondary folds. These programs are based on finding the secondary structure with the total lowest free energy by calculating the free energy of a number of base-pairing schemes and returning the lowest energy potential secondary structure as the most probable [11,12]. In the majority of cases, even for long sequences, the predicted structure is a structure whose tree representation is a small ordered tree (a tree with fewer than 10 vertices). However, there are secondary RNA structures whose tree representation is a tree with 10 or more vertices. For example, the 5S ribosomal RNA Clavibacter michiganensis (RNA Database ID S73542) has a 10 vertex tree representation.

### Research description

In this work we consider the possibility that a larger secondary RNA structure is formed by the bonding of two smaller secondary RNA structures. We model this bonding process by defining a graph merge that occurs on the vertices of the trees. If our hypothesis is valid, then larger secondary RNA structures should arise from trees that are unique to secondary RNA structure, and not from arbitrary trees. That is, only trees that represent RNA, and hence are thermodynamically stable structures, can be used to produce a tree which is still stable. We test this hypothesis and find, under specified constraints, stable trees are produced by merging two stable trees. Furthermore, by applying a predictive model, we find that some of the trees in the RAG database that are listed as candidate RNA structures are not clearly RNA-like in structure by our method. Our approach is novel, and may be considered as a valuable tool for refining prediction algorithms. It also illustrates the applicability of graphs as models, not only for secondary RNA, but for biomolecules in general. In order to formalize this idea, we introduce the graph-theoretic terminology and concepts.

### Basic terminology of Graph Theory

In graph theory, trees have been heavily studied both for application purposes and theoretical investigations. As defined in [13], a graph *G* = (*V*(*G*),* E*(*G*)) is a nonempty, finite set of elements called *vertices* together with a (possibly empty) set of unordered pairs of distinct vertices of *G* called* edges.* The *vertex set* of *G* is denoted by *V*(*G*), and the edge set of *G* is denoted by *E*(*G*). Here we consider only simple graphs, that is, graphs with no loops or multiple edges. A* tree* is commonly defined as a connected graph with the property that no two vertices lie on a cycle. These two properties, connected and acyclic, completely characterize a tree since the removal of any edge will disconnect the graph, and the addition of any edge will create a cycle. Further, this implies that any tree with *n* vertices contains* n* – 1 edges.

**Figure 1:**
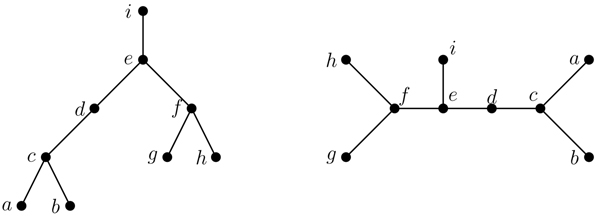
An example of two isomorphic trees

**Figure 2:**
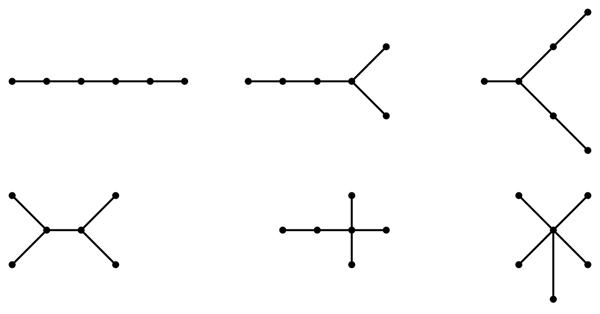
The six non-isomorphic trees of order 6

**Figure 3:**
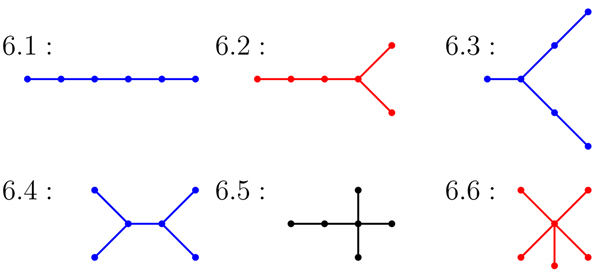
The index value and color category for trees of order 6 from RAG

An* isomorphism of graphs G and H* is a bijection between the vertex sets of *G* and* H*, *f* : *V*(*G*) → *V*(*H*), such that any two vertices *v* and *w* of *G* are adjacent in *G* if and only if *f* (*v*) and *f* (*w*) are adjacent in *H*. This kind of bijection is commonly called an* edge-preserving bijection* or a* structure-preserving bijection.* If an isomorphism exists between two graphs, then we say the graphs are* isomorphic* and we write *G* ≃ *H*. To illustrate these terms, Figure 1 displays two isomorphic trees. Figure 2 shows the six non-isomorphic trees of order 6. Figure 3 shows the index value and color codes of the six trees on 6 vertices as shown in [14]. Two vertices joined by an edge are said to be neighbors and the degree of a vertex *v* in a graph *G*, denoted by* deg_G_*(*v*), is the number of neighbors of *v* in *G*. A vertex of degree one is called a* leaf,* and its neighbor is called a* support vertex.* For use in this paper, a vertex *v* in a tree* T* is an* internal vertex* if it is neither a leaf or support vertex.

**Figure 4:**
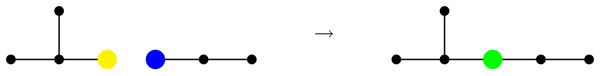
An example of a merge of two trees

Two vertices *u* and *v* are said to be* identified* if they are combined into a single vertex whose neighborhood is the union of the neighborhoods of *u* and *v*. The binary operation* merge* of two graphs *G*_1_ and *G*_2_ forms a new graph* G_uv_* by identifying a vertex *u* in *G*_1_ with a vertex *v* in *G*_2_. Figure 4 demonstrates vertex identification at the colored vertices for the pictured trees.

## Results and discussion

We consider the possibility that a larger secondary RNA structure could be formed by the bonding of two smaller secondary RNA structures. We model this bonding process by defining a merge operation on two trees. In this research, we determined all possible tree merges which result in a tree with 9 or fewer vertices. We use the RNA online-database RAG and the tree color code developed by Schlick et al. in [7] and discussed in the introduction. Recall that red trees are RNA-like (known), blue trees are RNA-like (candidates) and black trees are not RNA-like (non-candidates). Note that in a tree model of a secondary RNA structure, a hairpin corresponds to a vertex of degree one, internal loops and bulges are vertices of degree two, and junctions correspond to vertices of degree three or more.

Initially, we hypothesized that the color of the merging trees would be indicative of the color of the result tree. However, we found that this is not necessarily the case. Our hypothesis held when merging RNA-like tree motifs at vertices of degree one (hairpins) or degree two (bulges or internal loops). That is, when identifying vertices of degree two or less, almost all red to red or red to blue tree merges produced a red tree. However, this was not always the case when the vertices being identified included a vertex of high degree (junction).

Using these findings, we trained a neural network to recognize the known classification of a tree as RNA-like or not RNA-like in structure. The network assigned a value between 0 and 1 to classify these trees. Table 1 shows the interval values used to classify the trees as RNA-like or not-RNA-like. Paralleling the work completed in [15], our artificial neural network was trained on two classes of trees: the known RNA (red) trees and the non-candidate (black) trees. There are 15 red trees of order 7, 8 and 9 along with 11 black trees of order 7 and 8.

**Table 1:** The key for categorizing the artificial neural network prediction values

ANN Value	Resulting Category
1.0 - 0.80:	Highly-RNA-Like
	RNA-Like
	Unclassifiable
	Not-RNA-Like
	Highly Not-RNA-Like

The training and the predictions of the neural network were analyzed using standard methods in machine learning. To ameliorate overfitting, neural network training used leave-v-out cross-validation [16]. This involves partitioning the data into a majority training set and a minority complement (approximately 10% of the data in this effort). As the network is trained on the training set, its performance is assessed on the complement. In each of the 5 repetitions of the classifier experiment, the root-mean-square error for the complement predictions was less than 5% of the class value of 1 (i.e., below 0.05). In addition, the predictions were assessed using a Receiver Operating Characteristic(ROC), in which the true positive rate (sensitivity) is a function of the false positive rate (1 - specificity) for a binary classifier system as its discrimination threshold is varied [17]. Specifically, the area under the ROC curve (AUC) is equal to the probability that a classifier will rank a randomly chosen positive instance higher than a randomly chosen negative one. The AUC for the predictions ROC curve is 0.985.

### Predictions for the classified RAG trees

The MLP artificial neural network correctly predicted 100% of the known RAG trees to have a value greater than 0.50. Further, the network correctly calculated a prediction value below 0.50 for nine of the eleven black trees. However, two non-candidate RAG trees, indexed as 7.10 and 8.14, received an MLP prediction value between 0.60 and 0.50. Therefore, we label all trees with an MLP prediction value within the range  to .40 as “Unclassifiable”. Table 2 displays the RAG classification and corresponding predicted class for each of the classified 26 trees on 7, 8 or 9 vertices.

**Table 2:** The prediction values for the classified RAG trees

RAG Index	Color Class	ANN Prediction	ANN Result	RAG Index	Color Class	ANN Prediction	ANN Result
7.1	Red	1.00000	Highly RNA-Like	8.19	Black	0.33309	Not-RNA-Like
7.10	Black	0.59091	Unclassifiable	8.20	Red	0.69128	RNA-Like
7.11	Black	0.00045	Highly Not-RNA-Like	8.21	Black	0.00747	Highly Not-RNA-Like
7.2	Red	0.99860	Highly RNA-Like	8.22	Black	0.00437	Highly Not-RNA-Like
7.3	Red	0.99990	Highly RNA-Like	8.23	Black	0.00001	Highly Not-RNA-Like
7.6	Red	0.99721	Highly RNA-Like	8.3	Red	0.99796	Highly RNA-Like
7.9	Black	0.43130	Unclassifiable	8.5	Red	0.99991	Highly RNA-Like
8.10	Red	0.99994	Highly RNA-Like	8.7	Red	0.99815	Highly RNA-Like
8.11	Red	0.99682	Highly RNA-Like	8.9	Black	0.75935	RNA-Like
8.14	Black	0.52998	Unclassifiable	9.6	Red	0.99991	Highly RNA-Like
8.15	Red	0.56343	Unclassifiable	9.11	Red	0.99993	Highly RNA-Like
8.17	Black	0.36524	Not-RNA-Like	9.13	Red	0.99740	Highly RNA-Like
8.18	Black	0.00595	Highly Not-RNA-Like	9.27	Red	0.99795	Highly RNA-Like

### Predictions for the unclassified RAG trees

After using the MLP to predict the classified RAG trees, we calculated the prediction value for the 43 unclassified trees on 9 vertices from the RAG online web database. For these 43 trees, the MLP predicts a total of 22 trees to represent RNA motifs: 18 trees are highly-RNA-like and four are only RNA-like. Further, there are 14 trees which the artificial neural network predicts to not represent RNA secondary structure: 10 trees are highly not-RNA-like with four trees grouping into the not-RNA-like category. Overall, the MLP calculated an unclassifiable value for seven of the 43 trees. These values are listed in Table 3.

**Table 3:** The prediction values for the unclassified RAG trees

RAG Index	Color Class	ANN Prediction	ANN Result	RAG Index	Color Class	ANN Prediction	ANN Result
9.1	Unknown	1.00000	Highly RNA-Like	9.32	Unknown	0.00464	Highly Not-RNA-Like
9.10	Unknown	0.99874	Highly RNA-Like	9.33	Unknown	0.99897	Highly RNA-Like
9.12	Unknown	0.99724	Highly RNA-Like	9.34	Unknown	0.81259	Highly RNA-Like
9.14	Unknown	0.99722	Highly RNA-Like	9.35	Unknown	0.25818	Not-RNA-Like
9.15	Unknown	0.48019	Unclassifiable	9.36	Unknown	0.39903	Not-RNA-Like
9.16	Unknown	0.58718	Unclassifiable	9.37	Unknown	0.11790	Highly Not-RNA-Like
9.17	Unknown	0.99660	Highly RNA-Like	9.38	Unknown	0.57340	Unclassifiable
9.18	Unknown	0.99993	Highly RNA-Like	9.39	Unknown	0.00576	Highly Not-RNA-Like
9.19	Unknown	0.81428	Highly RNA-Like	9.4	Unknown	0.99840	Highly RNA-Like
9.2	Unknown	0.99888	Highly RNA-Like	9.40	Unknown	0.00238	Highly Not-RNA-Like
9.20	Unknown	0.58342	Unclassifiable	9.41	Unknown	0.00025	Highly Not-RNA-Like
9.21	Unknown	0.64473	RNA-Like	9.42	Unknown	0.69128	RNA-Like
9.22	Unknown	0.28250	Not-RNA-Like	9.43	Unknown	0.00756	Highly Not-RNA-Like
9.23	Unknown	0.84883	Highly RNA-Like	9.44	Unknown	0.40451	Unclassifiable
9.24	Unknown	0.99705	Highly RNA-Like	9.45	Unknown	0.00441	Highly Not-RNA-Like
9.25	Unknown	0.62696	RNA-Like	9.46	Unknown	0.00434	Highly Not-RNA-Like
9.26	Unknown	0.99942	Highly RNA-Like	9.47	Unknown	0.00002	Highly Not-RNA-Like
9.28	Unknown	0.36067	Not-RNA-Like	9.5	Unknown	0.72341	RNA-Like
9.29	Unknown	0.83375	Highly RNA-Like	9.7	Unknown	0.99875	Highly RNA-Like
9.3	Unknown	0.99840	Highly RNA-Like	9.8	Unknown	0.99909	Highly RNA-Like
9.30	Unknown	0.52189	Unclassifiable	9.9	Unknown	0.55979	Unclassifiable
9.31	Unknown	0.00769	Highly Not-RNA-Like				

### A comparative analysis

A predictive tool based on domination parameters was used in [15] to classify the all trees on 7, 8 and 9 vertices. Here we compare our results to the original tree categories determined in [7] and to results found in [15]. Our comparison is summarized in Table 4.

**Table 4:** A comparative analysis of the predicted results between [15], [14] and this paper.

RAG Index	RAG Color Class	Domination Predicted Class	Bonding Predicted Class	RAG Index	RAG Color Class	Domination Predicted Class	Bonding Predicted Class
7.1	Red	RNA-Like	Highly-RNA-Like	8.17	Black	Not-RNA-Like	Not-RNA-Like
7.10	Black	Not-RNA-Like	Unclassifiable	8.18	Black	Not-RNA-Like	Highly-Not-RNA-Like
7.11	Black	Not-RNA-Like	Highly-Not-RNA-Like	8.19	Black	Not-RNA-Like	Not-RNA-Like
7.2	Red	RNA-Like	Highly-RNA-Like	8.20	Red	RNA-Like	RNA-Like
7.3	Red	RNA-Like	Highly-RNA-Like	8.21	Black	Not-RNA-Like	Highly-Not-RNA-Like
7.6	Red	RNA-Like	Highly-RNA-Like	8.22	Black	Not-RNA-Like	Highly-Not-RNA-Like
7.9	Black	Not-RNA-Like	Unclassifiable	8.23	Black	Not-RNA-Like	Highly-Not-RNA-Like
8.10	Red	RNA-Like	Highly-RNA-Like	8.3	Red	RNA-Like	Highly-RNA-Like
8.11	Red	RNA-Like	Highly-RNA-Like	8.5	Red	RNA-Like	Highly-RNA-Like
8.14	Black	Not-RNA-Like	Unclassifiable	8.7	Red	RNA-Like	Highly-RNA-Like
8.15	Red	RNA-Like	Unclassifiable	8.9	Black	Not-RNA-Like	RNA-Like

9.1	Unkwn	RNA-Like	Highly-RNA-Like	9.31	Unkwn	RNA-Like	Highly-Not-RNA-Like
9.10	Unkwn	RNA-Like	Highly-RNA-Like	9.32	Unkwn	Not-RNA-Like	Highly-Not-RNA-Like
9.11	Red	RNA-Like	Highly-RNA-Like	9.33	Unkwn	RNA-Like	Highly-RNA-Like
9.12	Unkwn	RNA-Like	Highly-RNA-Like	9.34	Unkwn	RNA-Like	Highly-RNA-Like
9.13	Red	RNA-Like	Highly-RNA-Like	9.35	Unkwn	Not-RNA-Like	Not-RNA-Like
9.14	Unkwn	RNA-Like	Highly-RNA-Like	9.36	Unkwn	Not-RNA-Like	Not-RNA-Like
9.15	Unkwn	Not-RNA-Like	Unclassifiable	9.37	Unkwn	Not-RNA-Like	Highly-Not-RNA-Like
9.16	Unkwn	RNA-Like	Unclassifiable	9.38	Unkwn	RNA-Like	Unclassifiable
9.17	Unkwn	RNA-Like	Highly-RNA-Like	9.39	Unkwn	Not-RNA-Like	Highly-Not-RNA-Like
9.18	Unkwn	RNA-Like	Highly-RNA-Like	9.4	Unkwn	RNA-Like	Highly-RNA-Like
9.19	Unkwn	RNA-Like	Highly-RNA-Like	9.40	Unkwn	Not-RNA-Like	Highly-Not-RNA-Like
9.2	Unkwn	RNA-Like	Highly-RNA-Like	9.41	Unkwn	Not-RNA-Like	Highly-Not-RNA-Like
9.20	Unkwn	RNA-Like	Unclassifiable	9.42	Unkwn	RNA-Like	RNA-Like
9.21	Unkwn	RNA-Like	RNA-Like	9.43	Unkwn	RNA-Like	Highly-Not-RNA-Like
9.22	Unkwn	RNA-Like	Not-RNA-Like	9.44	Unkwn	RNA-Like	Unclassifiable
9.23	Unkwn	Not-RNA-Like	Highly-RNA-Like	9.45	Unkwn	Not-RNA-Like	Highly-Not-RNA-Like
9.24	Unkwn	RNA-Like	Highly-RNA-Like	9.46	Unkwn	Not-RNA-Like	Highly-Not-RNA-Like
9.25	Unkwn	Not-RNA-Like	RNA-Like	9.47	Unkwn	Not-RNA-Like	Highly-Not-RNA-Like
9.26	Unkwn	RNA-Like	Highly-RNA-Like	9.5	Unkwn	RNA-Like	RNA-Like
9.27	Red	RNA-Like	Highly-RNA-Like	9.6	Red	RNA-Like	Highly-RNA-Like
9.28	Unkwn	Not-RNA-Like	Not-RNA-Like	9.7	Unkwn	RNA-Like	Highly-RNA-Like
9.29	Unkwn	RNA-Like	Highly-RNA-Like	9.8	Unkwn	RNA-Like	Highly-RNA-Like
9.3	Unkwn	RNA-Like	Highly-RNA-Like	9.9	Unkwn	Not-RNA-Like	Unclassifiable
9.30	Unkwn	RNA-Like	Unclassifiable				

All three studies agreed on the classification of nine of the eleven non-candidate (black) tree graphs. The two exceptions are graphs 7.9 and 7.10, which our study finds to be unclassifiable. Further, with the exception of tree 8.15, all three research studies concluded that all known (red) tree graphs on 7, 8 and 9 vertices were RNA like based on their respective structural calculations. The model in [15] predicted tree 8.15 to be RNA-like in structure, however, their predictive model reported the highest amount of error for the classifications of this tree. We calculated a 0.56 likelihood that tree 8.15 contains RNA-like structure. As a result, both predictive models were unable to confidently classify tree 8.15.

Most notably, the predictive model used in previous RNA motif research supports the major results of this paper. As seen in Table 4, the predictive model in [15] classified 29 of the 43 unknown RNA trees to be RNA-like. When examining these results, the authors of [15] felt their model over predicted the class of RNA-like tree graphs. Accordingly, we found a total of 18 trees to be highly-RNA-like in structure. Of those, 17 of the 18 trees in the highly-RNA-like category from this study are included to be RNA-like from the results compiled by [15]. Consequently, the predictive model of our study narrows the class of RNA-like motifs from previous findings.

Additionally, of the 12 trees on 9 vertices that we predicted to be not-RNA-like in structure from our model, previous findings agreed with 10 of those classifications. The model in [15] predicted trees 9.31 and 9.43 to be RNA-like, whereas we found both motifs to be highly not-RNA-like. From the other direction, of the 14 trees predicted in [15] to not-RNA-like in structure, our prediction agreed with 12 of the 14. Trees 9.23 and 9.25 are both predicted to be not RNA-like in [15], but were classified as potential RNA structures in our study. Hence, our predictive model provides more descriptive information about the structural classification of the unknown RAG tree motifs on 9 vertices than the findings from [15]. In summary, when comparing our results with those in [15], we highlight two improvements. First, our neural network outcomes were not solely RNA-like or not RNA-like. Rather, our model assigns a probability, which is a measurement of a tree's RNA likeness. Second, our model predicts fewer of the trees on 9 vertices to be RNA-like, and thus seems to be a more discriminating predictive tool.

## Methods

We use graph theory to model the bonding of secondary RNA structures and a predictive neural network to quantify our results.

### Graph theoretic model

The binary operation* merge* of two trees *T*_1_ and *T*_2_ forms a new larger tree *T_uv_* by identifying a vertex *u* in *T*_1_ with a vertex *v* in *T*_2_. Merging two trees of *n* and *m* vertices produces *nm* total trees, some of which can be isomorphic, and each resulting tree has a total of *n* + *m* − 1 vertices.

**Figure 5:**
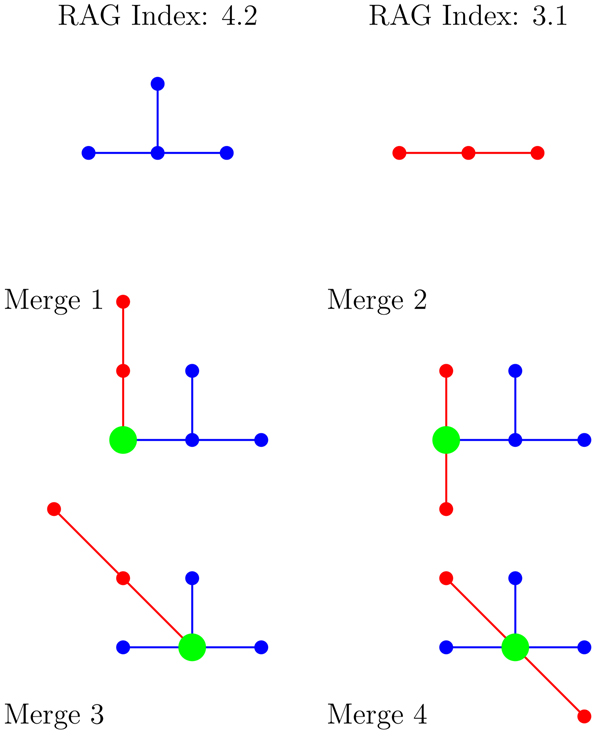
The four non-isomorphic resulting trees when merging trees 3.1 and 4.2

**Figure 6:**
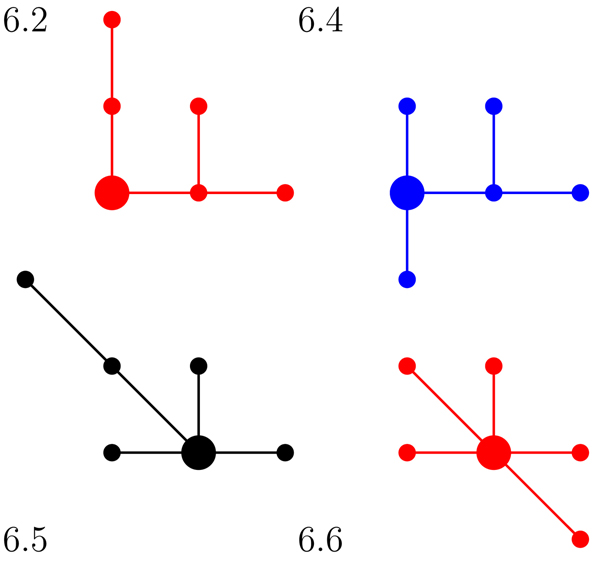
The RAG identification and color classes for the trees from Figure 5

To accurately model RNA bonding, we must consider all possible vertex identifications between two RNA tree models. For example, there are 12 possible vertex identifications for merging trees 3.1 and 4.2. Of these 12 merges, the four non-isomorphic trees are shown in Figure 5. Figure 6 displays the official RAG identification and color classes for the trees from Figure 5.

Our research determined all possible merges forming trees on 9 and fewer vertices. When tracking the information from all possible vertex identifications between two trees, the resulting trees were noted and their frequencies counted. For example, in Figure 6, the merge of tree 3.1 and 4.2 results in the following tree set: 3.1 + 4.2 = {6.2, 6.4, 6.5, 6.6}. Trees 6.2, 6.4, 6.5 and 6.6 occurred with frequencies 6, 3, 2, and 1 respectively. Additionally, we noted the type and degree of the vertices at each merge. Table 5 displays all the information for the vertex identifications of the merges between trees 3.1 and 4.2.

**Table 5:** The data table produced from the tree merge between RAG Trees 3.1 and 4.2

3.1 and 4.2	*v* ∊ *V* (3.1)		*v* ∊ *V* (4.2)		Results			Total Graphs
	
Merge:	*deg*(*v*)	Type	*deg*(*u*)	Type	Graph	Color		
1	1	Leaf	1	Leaf	6.2	Red		6
2	2	Support	1	Leaf	6.4	Blue		3
3	1	Leaf	3	Support	6.5	Black		2
4	2	Support	3	Support	6.6	Red		1

For all 94 graphs on 2 through 9 vertices, every possible vertex identification resulting in a graph on 9 or fewer vertices was calculated and recorded. Additional file 1 displays the vertex identification results for all tree merges. Then information from Additional file 1 was translated into data vectors. Each data vector displays the composition information for the result tree in the following manner:

[〈*c*_1_, *c*_2_, *deg*(*v*_1_), *deg*(*v*_2_)〉, 〈*y*_1_, *y*_2_〉], where for *i*  ∊ {1, 2},

*deg*(*v_i_*) is the degree of the identified vertex of *T_i_*, and

*y*_1_ = 1 and *y*_2_ = 0 if the result tree is an RNA-like tree, and *y*_1_ = 0 and* y*_2_ = 1 if the result tree is not RNA-like.

### An artificial neural network

In their numerical form as data vectors, the vertex identification results are used to predict the RNA-like status of the 43 unclassified trees on 9 vertices. The data vectors from the 15 known (red) tree graphs on 7, 8 and 9 vertices along with the data vectors of the 11 non-candidate (black) tree graphs on 7 and 8 vertices make up the training data. To check the validity of our model, we then predicted the status of the 26 known tree classifications on 7, 8 and 9 vertices. Then, the model is used as a predictive tool for the 43 unclassified trees of order nine. This research parallels previous work by the authors in [15]. In this section, we discuss the details of designing, training and using an artificial neural network as a prediction tool.

#### *Description*

Following the network created in [15], our approach is to train a multi-layer perceptron (MLP) artificial neural network using a standard back-propagation algorithm. Results from a back-propagation MLP can be reproduced independently by other researchers and can also provide information beyond simple predictions. The 3-layer MLP is used to predict the RNA-like status of the trees. The first layer, or* input later,* contains four perceptrons corresponding to the data vector from one vertex identification of the complete merge between two trees. The last layer, or* output layer,* consists of two perceptrons with activations *y*_1_ and *y*_2_, where *y*_1_ = 1 and *y*_2_ = 0 if the result tree, which corresponds to the input data vector, is predicted to be an RNA tree and where *y*_1_ = 0 and *y*_2_ = 1 if the result tree is not RNA-like. The middle layer, or *hidden layer,* is comprised of 24 perceptrons. The weights between the input and hidden layers will be denoted by *w _jk_* and the weights between the hidden and output layers will be denoted by *α_ij_*.

#### *Implementation*

The data vectors from the vertex identifications of the 26 trees on 7, 8 or 9 vertices that either are an RNA tree or not an RNA-like tree determine the training set

Where  is the data vector, *q^i^* = 〈1, 0〉 if the tree is known or predicted to be an RNA tree, and *q^i^* = 〈0, 1〉 of the tree is not RNA-like. The back-propagation algorithm is used to implement a gradient following minimization of the total squared error

where *y*(*p^i^*) = 〈*y*_1_(*p^i^*), *y*_2_(*p^i^*)〉 is the output due to an input of *p^i^* and the norm is generated by the corresponding dot product.

The weights are initially assigned random values close to 0. Then, for each pair (*p^i^*, *q^i^*) the weights *α_jk_* are adjusting using

*α_jk_* → *α_jk_* + λ*δ_j_*ξ*_k_*

where  where λ > 0 is a fixed parameter called the* learning rate,* and where

The weights *ω_kr_* are adjusted using

In each training session, the patterns should be randomly permuted to avoid bias, and training should continue until *E* is sufficiently close to 0 [18].

The MLP artificial neural network was trained and tested by* predicting complements.* During this procedure, the vertex identification data vectors of the 26 classified tree motifs were randomly partitioned into a training set and a complement set. Predicting complements was performed with 15% of the data vectors in the complement set for each trial.

The network was trained using the data* not in* the complement until the total squared error was close to 0 (approximately 10,000 iterations for each of the 5 repetitions of the classifier experiment). Once the network is trained, it is used to predict the classification of the data in the complement set. This is known as leave-v-out cross-validation. According to [16], cross-validation is a reliable measure of the generalization error of the network when the training set is not too large.

In order to most accurately utilize our data, each tree’s final classification was calculated as an average of a linear combination of prediction values from the vertex identifications. To do so, we began this procedure by using the MLP to predict the value for each vertex identification for a given tree. Then, this value was multiplied by a weight which refers to the total number of graph isomorphisms for the vertex identification. This weight was noted for each identification and can be referenced in the “Total Graphs” column of Additional file 1. To normalize the result, the linear combination of all the vertex identification values was divided by the sum of the weights. This final average determined the prediction value for the tree. Table 6 outlines this procedure for tree 7.9.

**Table 6:** An example of the algorithm used to determine the prediction value for tree 7.9

*a*[7.9] : = 4 * *RNANet* : *–Classify* (〈1, 1, 1, 3〉);		*a*_[7.9]_ : = 〈0.95945, 3.52754〉
*b*[7.9] : = 2 * *RNANet* : *–Classify* (〈1, 0, 1, 2〉);		*b*_[7.9]_ : = 〈0.00030, 1.99985〉
*c*[7.9] : = 1 * *RNANet* : *–Classify* (〈1, 1, 2, 2〉);		*c*_[7.9]_ : = 〈0.97666, 0.02253〉
*d*[7.9] : = 4 * *RNANet* : *–Classify* (〈1, 1, 2, 1〉);		*d*_[7.9]_ : = 〈3.95652, 0.05185〉
*e*[7.9] : = 6 * *RNANet* : *–Classify* (〈1, 1, 1, 3〉);		*e*_[7.9]_ : = 〈1.43917, 5.29130〉
		*Class*_[7.9]_ := 〈0.43130, 0.64077〉

**Figure 7:**
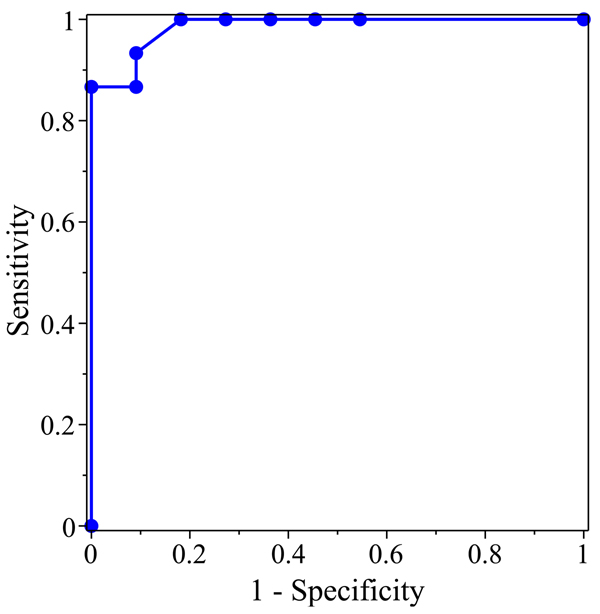
The Receiver Operating Characteristic for the Neural Network Predictions of the classified RAG trees

Once the MLP was fully trained, the network was used to predict the classification of the 26 red or black RAG trees on 7, 8 and 9 vertices. For these trees, the final MLP prediction values range from 1.0 to 0.0. As a result, the key in Table 1 uses a range to classify the final values. The ROC curve for the results in Table 2 are shown in Figure 7. It is worth noting that ROC analysis suggests that 0.6 is the “best threshold” between RNA-like and not RNA-like, thus supporting the use of the key in Table 1.

## Conclusions

Using a tree representation of secondary RNA structure, we model the creation of a larger structure from the bonding of two smaller structures by considering all combinatorial possibilities. We model the bonding with a graph operation called (vertex) merge. Data from this process included information on the degrees of the vertices identified in the merge and the classification of substructures. We created data vectors and then utilized these data vectors from known RNA trees on 7, 8 and 9 vertices together with the data vectors from the non-candidate RNA trees on 7 and 8 vertices to create and train a neural network to recognize a tree as RNA-like or not-RNA-like in structure. We applied this predictive tool to categorize known RNA classifications and to predict unknown RNA trees.

The results for the 15 red trees of orders seven, eight, and nine agree with the classifications from the RAG database and previous research in [15]. Further, our neural network correctly classified 9 of the 11 black, or non-candidate, trees on 7 and 8 vertices to agree with previous research. However, the authors of [15] felt their model over predicted the class of RNA-like trees for those 43 unclassified trees on 9 vertices. Their results classified 29 of the unknown trees as RNA-like in structure, and 14 as not-RNA-like. As a result, this study narrows down the class of highly-RNA-like tree structures on 9 vertices from the 29 predicted in [15] to 18 according to the values calculated by the MLP artificial neural network.

We revealed that graphical operations from the field of mathematical graph theory can successfully model secondary RNA motifs. Further, we demonstrated that these numerical values from these operations can enable the training of an artificial neural network to recognize the difference between likely and unlikely RNA structures. These findings, along with those from previous predictive models, exhibit the power of mathematical graph theory as an effective modeling method. By representing bimolecular structures with graph theory, modern researchers enter an extensive and unexplored field of quantitative biology. Although trees have previously been used to model secondary RNA structure, the applications of techniques from graph theory have been limited. There are numerous binary operations on graphs, such as the Cartesian product and graph join. In this paper, we have introduced graph merge as a novel approach to the study of RNA binding.

As a follow up to this study, future research could combine the data from [7,15] and this paper to create a more powerful predictive model. A more intelligent artificial neural network, or another predictive tool, could utilize all three sets of data to predict the classifications for all the RAG trees. Additionally, future projects could examine the effect of other graphical invariants and operations on the structural properties of the RAG motifs. Another potential research project could be to use the ideas of our research, and those from [15], to examine the structural components of the unclassified RAG trees on 10 vertices from [14].

## Competing interests

The authors declare that they have no competing interests.

## Authors' contributions

DRK produced the tree merge data, trained the neural network, and contributed to writing the paper. DJK conceived of the study, participated in its coordination and execution, and was involved in drafting the manuscript and revising it critically for important intellectual content. JK designed and helped train the neural network. TH participated in the coordination of the study, helped with the graph theory, and was involved in drafting the manuscript. All authors read and approved the final manuscript.

## Supplementary Material

Additional file 1 Click here for file
