# Supplementary material for: A predictive model for secondary RNA structure using graph theory and a neural network
Source: BMC Bioinformatics. 2010 Oct 7;11(Suppl 6):S21. doi: 10.1186/1471-2105-11-S6-S21 (PMC3026369; doi:10.1186/1471-2105-11-S6-S21)
Supplement: Additional file 1 [file 1471-2105-11-S6-S21-S1.pdf]

Table A - The complete table of vertex identifications from all tree merges

RAG Trees to be Merged	DATA FROM THE 1st TREE			DATA FROM THE 2nd TREE			RESULTS		
	Tree Color	Vert. Type	deg (v)	Tree Color	Vert. Type	deg (v)	RAG Graph	Tree Color	Total Graphs
2.1+2.1	Red	Leaf	1	Red	Leaf	1	3.1	Red	4
2.1+3.1	Red	Leaf	1	Red	Leaf	1	4.1	Red	4
2.1+3.1	Red	Leaf	1	Red	Support	2	4.2	Blue	2
2.1+4.1	Red	Leaf	1	Red	Leaf	1	5.1	Red	4
2.1+4.1	Red	Leaf	1	Red	Support	2	5.2	Red	4
2.1+4.2	Red	Leaf	1	Blue	Leaf	1	5.2	Red	6
2.1+4.2	Red	Leaf	1	Blue	Support	3	5.3	Red	2
2.1+5.1	Red	Leaf	1	Red	Leaf	1	6.1	Blue	4
2.1+5.1	Red	Leaf	1	Red	Support	2	6.2	Red	4
2.1+5.1	Red	Leaf	1	Red	Internal	2	6.3	Blue	2
2.1+5.2	Red	Leaf	1	Red	Leaf	1	6.2	Red	2
2.1+5.2	Red	Leaf	1	Red	Leaf	1	6.3	Blue	4
2.1+5.2	Red	Leaf	1	Red	Support	2	6.4	Blue	2
2.1+5.2	Red	Leaf	1	Red	Support	3	6.5	Black	2
2.1+5.3	Red	Leaf	1	Red	Leaf	1	6.5	Black	8
2.1+5.3	Red	Leaf	1	Red	Support	4	6.6	Red	2
2.1+6.1	Red	Leaf	1	Blue	Leaf	1	7.1	Red	4
2.1+6.1	Red	Leaf	1	Blue	Support	2	7.2	Red	4
2.1+6.1	Red	Leaf	1	Blue	Internal	2	7.3	Red	4
2.1+6.2	Red	Leaf	1	Red	Leaf	1	7.2	Red	2
2.1+6.2	Red	Leaf	1	Red	Leaf	1	7.3	Red	4
2.1+6.2	Red	Leaf	1	Red	Support	2	7.4	Blue	2
2.1+6.2	Red	Leaf	1	Red	Support	3	7.5	Blue	2
2.1+6.2	Red	Leaf	1	Red	Internal	2	7.6	Red	2
2.1+6.3	Red	Leaf	1	Blue	Leaf	1	7.3	Red	4
2.1+6.3	Red	Leaf	1	Blue	Support	2	7.6	Red	4
2.1+6.3	Red	Leaf	1	Blue	Support	3	7.7	Blue	2
2.1+6.3	Red	Leaf	1	Blue	Leaf	1	7.8	Blue	2
2.1+6.4	Red	Leaf	1	Blue	Leaf	1	7.6	Red	8
2.1+6.4	Red	Leaf	1	Blue	Support	3	7.9	Black	4
2.1+6.5	Red	Leaf	1	Black	Support	4	7.10	Black	2
2.1+6.5	Red	Leaf	1	Black	Leaf	1	7.5	Blue	2
2.1+6.5	Red	Leaf	1	Black	Leaf	1	7.7	Blue	6
2.1+6.5	Red	Leaf	1	Black	Support	2	7.9	Black	2
2.1+6.6	Red	Leaf	1	Red	Leaf	1	7.10	Black	10
2.1+6.6	Red	Leaf	1	Red	Support	5	7.11	Black	2
2.1+7.1	Red	Leaf	1	Red	Leaf	1	8.1	Blue	4
2.1+7.1	Red	Leaf	1	Red	Support	2	8.2	Blue	4

RAG Trees to be Merged	DATA FROM THE 1st TREE			DATA FROM THE 2nd TREE			RESULTS		
	Tree Color	Vert. Type	*deg* (v)	Tree Color	Vert. Type	*deg* (v)	RAG Graph	Tree Color	Total Graphs
2.1+7.1	Red	Leaf	1	Red	Internal	2	8.3	Red	4
2.1+7.1	Red	Leaf	1	Red	Internal	2	8.5	Red	2
2.1+7.10	Red	Leaf	1	Black	Leaf	1	8.14	Black	2
2.1+7.10	Red	Leaf	1	Black	Support	2	8.19	Black	2
2.1+7.10	Red	Leaf	1	Black	Leaf	1	8.21	Black	8
2.1+7.10	Red	Leaf	1	Black	Support	5	8.22	Black	2
2.1+7.11	Red	Leaf	1	Black	Leaf	1	8.22	Black	12
2.1+7.11	Red	Leaf	1	Black	Support	6	8.23	Black	2
2.1+7.2	Red	Leaf	1	Red	Leaf	1	8.2	Blue	2
2.1+7.2	Red	Leaf	1	Red	Leaf	1	8.3	Red	4
2.1+7.2	Red	Leaf	1	Red	Support	2	8.4	Blue	2
2.1+7.2	Red	Leaf	1	Red	Support	3	8.6	Blue	2
2.1+7.2	Red	Leaf	1	Red	Internal	2	8.7	Red	2
2.1+7.2	Red	Leaf	1	Red	Internal	2	8.8	Blue	2
2.1+7.3	Red	Leaf	1	Red	Leaf	1	8.10	Red	2
2.1+7.3	Red	Leaf	1	Red	Internal	2	8.11	Red	2
2.1+7.3	Red	Leaf	1	Red	Internal	3	8.12	Blue	2
2.1+7.3	Red	Leaf	1	Red	Leaf	1	8.3	Red	2
2.1+7.3	Red	Leaf	1	Red	Leaf	1	8.5	Red	2
2.1+7.3	Red	Leaf	1	Red	Support	2	8.7	Red	2
2.1+7.3	Red	Leaf	1	Red	Support	2	8.8	Blue	2
2.1+7.4	Red	Leaf	1	Blue	Internal	2	8.13	Blue	2
2.1+7.4	Red	Leaf	1	Blue	Support	3	8.9	Black	4
2.1+7.5	Red	Leaf	1	Blue	Leaf	1	8.12	Blue	6
2.1+7.5	Red	Leaf	1	Blue	Support	4	8.14	Black	2
2.1+7.5	Red	Leaf	1	Blue	Internal	2	8.15	Red	2
2.1+7.5	Red	Leaf	1	Blue	Leaf	1	8.6	Blue	2
2.1+7.5	Red	Leaf	1	Blue	Support	2	8.9	Black	2
2.1+7.6	Red	Leaf	1	Red	Leaf	1	8.11	Red	4
2.1+7.6	Red	Leaf	1	Red	Support	2	8.13	Blue	2
2.1+7.6	Red	Leaf	1	Red	Support	3	8.15	Red	2
2.1+7.6	Red	Leaf	1	Red	Leaf	1	8.16	Blue	2
2.1+7.6	Red	Leaf	1	Red	Internal	3	8.17	Black	2
2.1+7.6	Red	Leaf	1	Red	Leaf	1	8.8	Blue	2
2.1+7.7	Red	Leaf	1	Blue	Leaf	1	8.12	Blue	4
2.1+7.7	Red	Leaf	1	Blue	Support	2	8.17	Black	4
2.1+7.7	Red	Leaf	1	Blue	Leaf	1	8.20	Red	4
2.1+7.7	Red	Leaf	1	Blue	Internal	4	8.21	Black	2
2.1+7.8	Red	Leaf	1	Blue	Leaf	1	8.10	Red	6
2.1+7.8	Red	Leaf	1	Blue	Support	2	8.16	Blue	6
2.1+7.8	Red	Leaf	1	Blue	Internal	3	8.20	Red	2
2.1+7.9	Red	Leaf	1	Black	Leaf	1	8.15	Red	4

RAG Trees to be Merged	DATA FROM THE 1st TREE			DATA FROM THE 2nd TREE			RESULTS		
	Tree Color	Vert. Type	*deg* (v)	Tree Color	Vert. Type	*deg* (v)	RAG Graph	Tree Color	Total Graphs
2.1+7.9	Red	Leaf	1	Black	Leaf	1	8.17	Black	6
2.1+7.9	Red	Leaf	1	Black	Support	3	8.18	Black	2
2.1+7.9	Red	Leaf	1	Black	Support	4	8.19	Black	2
2.1+8.1	Red	Leaf	1	Blue	Leaf	1	9.1	Unknown	4
2.1+8.1	Red	Leaf	1	Blue	Support	2	9.2	Unknown	4
2.1+8.1	Red	Leaf	1	Blue	Internal	2	9.4	Unknown	4
2.1+8.1	Red	Leaf	1	Blue	Internal	2	9.6	Red	4
2.1+8.10	Red	Leaf	1	Red	Leaf	1	9.11	Red	2
2.1+8.10	Red	Leaf	1	Red	Support	2	9.17	Unknown	2
2.1+8.10	Red	Leaf	1	Red	Leaf	1	9.18	Unknown	4
2.1+8.10	Red	Leaf	1	Red	Support	2	9.26	Unknown	4
2.1+8.10	Red	Leaf	1	Red	Internal	2	9.27	Red	2
2.1+8.10	Red	Leaf	1	Red	Internal	3	9.29	Unknown	2
2.1+8.11	Red	Leaf	1	Red	Leaf	1	9.14	Unknown	4
2.1+8.11	Red	Leaf	1	Red	Support	2	9.24	Unknown	4
2.1+8.11	Red	Leaf	1	Red	Leaf	1	9.27	Red	4
2.1+8.11	Red	Leaf	1	Red	Internal	3	9.30	Unknown	4
2.1+8.12	Red	Leaf	1	Blue	Leaf	1	9.19	Unknown	2
2.1+8.12	Red	Leaf	1	Blue	Leaf	1	9.21	Unknown	2
2.1+8.12	Red	Leaf	1	Blue	Support	2	9.22	Unknown	2
2.1+8.12	Red	Leaf	1	Blue	Leaf	1	9.29	Unknown	4
2.1+8.12	Red	Leaf	1	Blue	Internal	2	9.30	Unknown	2
2.1+8.12	Red	Leaf	1	Blue	Internal	4	9.31	Unknown	2
2.1+8.12	Red	Leaf	1	Blue	Support	2	9.44	Unknown	2
2.1+8.13	Red	Leaf	1	Blue	Leaf	1	9.24	Unknown	8
2.1+8.13	Red	Leaf	1	Blue	Support	3	9.28	Unknown	4
2.1+8.13	Red	Leaf	1	Blue	Leaf	1	9.33	Unknown	2
2.1+8.13	Red	Leaf	1	Blue	Support	3	9.35	Unknown	2
2.1+8.14	Red	Leaf	1	Black	Leaf	1	9.15	Unknown	2
2.1+8.14	Red	Leaf	1	Black	Support	2	9.25	Unknown	2
2.1+8.14	Red	Leaf	1	Black	Leaf	1	9.31	Unknown	8
2.1+8.14	Red	Leaf	1	Black	Support	2	9.32	Unknown	2
2.1+8.14	Red	Leaf	1	Black	Internal	2	9.36	Unknown	2
2.1+8.15	Red	Leaf	1	Red	Leaf	1	9.20	Unknown	2
2.1+8.15	Red	Leaf	1	Red	Support	2	9.28	Unknown	2
2.1+8.15	Red	Leaf	1	Red	Leaf	1	9.30	Unknown	6
2.1+8.15	Red	Leaf	1	Red	Leaf	1	9.34	Unknown	2
2.1+8.15	Red	Leaf	1	Red	Support	4	9.36	Unknown	2
2.1+8.15	Red	Leaf	1	Red	Internal	3	9.37	Unknown	2
2.1+8.16	Red	Leaf	1	Blue	Leaf	1	9.26	Unknown	4
2.1+8.16	Red	Leaf	1	Blue	Leaf	1	9.27	Red	4

RAG Trees to be Merged	DATA FROM THE 1st TREE			DATA FROM THE 2nd TREE			RESULTS		
	Tree Color	Vert. Type	*deg* (v)	Tree Color	Vert. Type	*deg* (v)	RAG Graph	Tree Color	Total Graphs
2.1+8.16	Red	Leaf	1	Blue	Support	2	9.33	Unknown	4
2.1+8.16	Red	Leaf	1	Blue	Support	3	9.34	Unknown	2
2.1+8.16	Red	Leaf	1	Blue	Internal	3	9.38	Unknown	2
2.1+8.17	Red	Leaf	1	Black	Leaf	1	9.30	Unknown	4
2.1+8.17	Red	Leaf	1	Black	Support	2	9.35	Unknown	2
2.1+8.17	Red	Leaf	1	Black	Support	3	9.37	Unknown	2
2.1+8.17	Red	Leaf	1	Black	Leaf	1	9.38	Unknown	4
2.1+8.17	Red	Leaf	1	Black	Internal	4	9.39	Unknown	2
2.1+8.17	Red	Leaf	1	Black	Leaf	1	9.44	Unknown	2
2.1+8.18	Red	Leaf	1	Black	Leaf	1	9.37	Unknown	12
2.1+8.18	Red	Leaf	1	Black	Support	4	9.40	Unknown	4
2.1+8.19	Red	Leaf	1	Black	Leaf	1	9.36	Unknown	4
2.1+8.19	Red	Leaf	1	Black	Leaf	1	9.39	Unknown	8
2.1+8.19	Red	Leaf	1	Black	Support	3	9.40	Unknown	2
2.1+8.19	Red	Leaf	1	Black	Support	5	9.41	Unknown	2
2.1+8.2	Red	Leaf	1	Blue	Internal	2	9.10	Unknown	2
2.1+8.2	Red	Leaf	1	Blue	Leaf	1	9.2	Unknown	2
2.1+8.2	Red	Leaf	1	Blue	Support	2	9.3	Unknown	2
2.1+8.2	Red	Leaf	1	Blue	Leaf	1	9.4	Unknown	4
2.1+8.2	Red	Leaf	1	Blue	Support	3	9.5	Unknown	2
2.1+8.2	Red	Leaf	1	Blue	Internal	2	9.7	Unknown	2
2.1+8.2	Red	Leaf	1	Blue	Internal	2	9.8	Unknown	2
2.1+8.20	Red	Leaf	1	Red	Leaf	1	9.29	Unknown	6
2.1+8.20	Red	Leaf	1	Red	Support	2	9.38	Unknown	6
2.1+8.20	Red	Leaf	1	Red	Leaf	1	9.42	Unknown	2
2.1+8.20	Red	Leaf	1	Red	Internal	4	9.43	Unknown	2
2.1+8.21	Red	Leaf	1	Black	Leaf	1	9.31	Unknown	4
2.1+8.21	Red	Leaf	1	Black	Support	2	9.39	Unknown	4
2.1+8.21	Red	Leaf	1	Black	Leaf	1	9.43	Unknown	6
2.1+8.21	Red	Leaf	1	Black	Support	5	9.45	Unknown	2
2.1+8.22	Red	Leaf	1	Black	Leaf	1	9.32	Unknown	2
2.1+8.22	Red	Leaf	1	Black	Support	2	9.41	Unknown	2
2.1+8.22	Red	Leaf	1	Black	Leaf	1	9.45	Unknown	10
2.1+8.22	Red	Leaf	1	Black	Internal	5	9.46	Unknown	2
2.1+8.23	Red	Leaf	1	Black	Leaf	1	9.46	Unknown	14
2.1+8.23	Red	Leaf	1	Black	Internal	7	9.47	Unknown	2
2.1+8.3	Red	Leaf	1	Red	Leaf	1	9.11	Red	2
2.1+8.3	Red	Leaf	1	Red	Internal	2	9.12	Unknown	2
2.1+8.3	Red	Leaf	1	Red	Internal	2	9.14	Unknown	2
2.1+8.3	Red	Leaf	1	Red	Support	3	9.21	Unknown	2
2.1+8.3	Red	Leaf	1	Red	Leaf	1	9.4	Unknown	2

RAG Trees to be Merged	DATA FROM THE 1st TREE			DATA FROM THE 2nd TREE			RESULTS		
	Tree Color	Vert. Type	*deg* (v)	Tree Color	Vert. Type	*deg* (v)	RAG Graph	Tree Color	Total Graphs
2.1+8.3	Red	Leaf	1	Red	Leaf	1	9.6	Red	2
2.1+8.3	Red	Leaf	1	Red	Support	2	9.7	Unknown	2
2.1+8.3	Red	Leaf	1	Red	Support	2	9.8	Unknown	2
2.1+8.4	Red	Leaf	1	Blue	Internal	2	9.13	Red	4
2.1+8.4	Red	Leaf	1	Blue	Leaf	1	9.7	Unknown	8
2.1+8.4	Red	Leaf	1	Blue	Support	3	9.9	Unknown	4
2.1+8.5	Red	Leaf	1	Red	Support	2	9.10	Unknown	4
2.1+8.5	Red	Leaf	1	Red	Internal	2	9.14	Unknown	4
2.1+8.5	Red	Leaf	1	Red	Leaf	1	9.18	Unknown	2
2.1+8.5	Red	Leaf	1	Red	Support	3	9.19	Unknown	2
2.1+8.5	Red	Leaf	1	Red	Leaf	1	9.6	Red	4
2.1+8.6	Red	Leaf	1	Blue	Support	4	9.15	Unknown	2
2.1+8.6	Red	Leaf	1	Blue	Internal	2	9.16	Unknown	2
2.1+8.6	Red	Leaf	1	Blue	Internal	2	9.20	Unknown	2
2.1+8.6	Red	Leaf	1	Blue	Leaf	1	9.21	Unknown	6
2.1+8.6	Red	Leaf	1	Blue	Leaf	1	9.5	Unknown	2
2.1+8.6	Red	Leaf	1	Blue	Support	2	9.9	Unknown	2
2.1+8.7	Red	Leaf	1	Red	Leaf	1	9.10	Unknown	2
2.1+8.7	Red	Leaf	1	Red	Leaf	1	9.12	Unknown	4
2.1+8.7	Red	Leaf	1	Red	Support	2	9.13	Red	2
2.1+8.7	Red	Leaf	1	Red	Support	3	9.16	Unknown	2
2.1+8.7	Red	Leaf	1	Red	Leaf	1	9.17	Unknown	2
2.1+8.7	Red	Leaf	1	Red	Internal	3	9.22	Unknown	2
2.1+8.7	Red	Leaf	1	Red	Internal	2	9.24	Unknown	2
2.1+8.8	Red	Leaf	1	Blue	Support	2	9.13	Red	2
2.1+8.8	Red	Leaf	1	Blue	Leaf	1	9.14	Unknown	4
2.1+8.8	Red	Leaf	1	Blue	Support	3	9.20	Unknown	2
2.1+8.8	Red	Leaf	1	Blue	Internal	2	9.24	Unknown	2
2.1+8.8	Red	Leaf	1	Blue	Leaf	1	9.26	Unknown	2
2.1+8.8	Red	Leaf	1	Blue	Internal	3	9.44	Unknown	2
2.1+8.8	Red	Leaf	1	Blue	Leaf	1	9.8	Unknown	2
2.1+8.9	Red	Leaf	1	Black	Leaf	1	9.16	Unknown	4
2.1+8.9	Red	Leaf	1	Black	Leaf	1	9.22	Unknown	6
2.1+8.9	Red	Leaf	1	Black	Support	3	9.23	Unknown	2
2.1+8.9	Red	Leaf	1	Black	Support	4	9.25	Unknown	2
2.1+8.9	Red	Leaf	1	Black	Internal	2	9.28	Unknown	2
3.1+3.1	Red	Leaf	1	Red	Leaf	1	5.1	Red	4
3.1+3.1	Red	Leaf	1	Red	Support	2	5.2	Red	4
3.1+3.1	Red	Support	2	Red	Support	2	5.3	Red	1
3.1+4.1	Red	Leaf	1	Red	Leaf	1	6.1	Blue	4
3.1+4.1	Red	Support	2	Red	Leaf	1	6.2	Red	2

RAG Trees to be Merged	DATA FROM THE 1st TREE			DATA FROM THE 2nd TREE			RESULTS		
	Tree Color	Vert. Type	*deg* (v)	Tree Color	Vert. Type	*deg* (v)	RAG Graph	Tree Color	Total Graphs
3.1+4.1	Red	Leaf	1	Red	Support	2	6.3	Blue	4
3.1+4.1	Red	Support	2	Red	Support	2	6.5	Black	2
3.1+4.2	Red	Leaf	1	Blue	Leaf	1	6.2	Red	6
3.1+4.2	Red	Support	2	Blue	Leaf	1	6.4	Blue	3
3.1+4.2	Red	Leaf	1	Blue	Support	3	6.5	Black	2
3.1+4.2	Red	Support	2	Blue	Support	3	6.6	Red	1
3.1+5.1	Red	Leaf	1	Red	Leaf	1	7.1	Red	4
3.1+5.1	Red	Support	2	Red	Leaf	1	7.2	Red	2
3.1+5.1	Red	Leaf	1	Red	Support	2	7.3	Red	4
3.1+5.1	Red	Support	2	Red	Support	2	7.5	Blue	2
3.1+5.1	Red	Support	2	Red	Internal	2	7.7	Blue	1
3.1+5.1	Red	Leaf	1	Red	Internal	2	7.8	Blue	2
3.1+5.2	Red	Support	2	Red	Support	3	7.10	Black	1
3.1+5.2	Red	Leaf	1	Red	Leaf	1	7.2	Red	2
3.1+5.2	Red	Leaf	1	Red	Leaf	1	7.3	Red	4
3.1+5.2	Red	Support	2	Red	Leaf	1	7.4	Blue	1
3.1+5.2	Red	Leaf	1	Red	Support	2	7.6	Red	4
3.1+5.2	Red	Leaf	1	Red	Support	3	7.7	Blue	2
3.1+5.2	Red	Support	2	Red	Support	2	7.9	Black	1
3.1+5.3	Red	Leaf	1	Red	Support	4	7.10	Black	2
3.1+5.3	Red	Support	2	Red	Support	4	7.11	Black	1
3.1+5.3	Red	Leaf	1	Red	Leaf	1	7.5	Blue	8
3.1+5.3	Red	Support	2	Red	Leaf	1	7.9	Black	4
3.1+6.1	Red	Leaf	1	Blue	Leaf	1	8.1	Blue	4
3.1+6.1	Red	Leaf	1	Blue	Internal	2	8.10	Red	4
3.1+6.1	Red	Support	2	Blue	Internal	2	8.12	Blue	2
3.1+6.1	Red	Support	2	Blue	Leaf	1	8.2	Blue	2
3.1+6.1	Red	Leaf	1	Blue	Support	2	8.3	Red	4
3.1+6.1	Red	Support	2	Blue	Support	2	8.6	Blue	2
3.1+6.2	Red	Leaf	1	Red	Internal	3	8.12	Blue	2
3.1+6.2	Red	Support	2	Red	Internal	3	8.14	Black	1
3.1+6.2	Red	Leaf	1	Red	Internal	2	8.16	Blue	2
3.1+6.2	Red	Support	2	Red	Internal	2	8.17	Black	1
3.1+6.2	Red	Leaf	1	Red	Leaf	1	8.2	Blue	2
3.1+6.2	Red	Support	2	Red	Leaf	1	8.4	Blue	1
3.1+6.2	Red	Leaf	1	Red	Leaf	1	8.5	Red	4
3.1+6.2	Red	Leaf	1	Red	Support	2	8.7	Red	2
3.1+6.2	Red	Support	2	Red	Leaf	1	8.8	Blue	2
3.1+6.2	Red	Support	2	Red	Support	2	8.9	Black	1
3.1+6.3	Red	Leaf	1	Blue	Leaf	1	8.10	Red	2
3.1+6.3	Red	Leaf	1	Blue	Support	2	8.11	Red	4

RAG Trees to be Merged	DATA FROM THE 1st TREE			DATA FROM THE 2nd TREE			RESULTS		
	Tree Color	Vert. Type	*deg* (v)	Tree Color	Vert. Type	*deg* (v)	RAG Graph	Tree Color	Total Graphs
3.1+6.3	Red	Leaf	1	Blue	Leaf	1	8.13	Blue	4
3.1+6.3	Red	Support	2	Blue	Support	2	8.15	Red	2
3.1+6.3	Red	Support	2	Blue	Leaf	1	8.16	Blue	1
3.1+6.3	Red	Leaf	1	Blue	Support	3	8.20	Red	2
3.1+6.3	Red	Support	2	Blue	Support	3	8.21	Black	1
3.1+6.3	Red	Support	2	Blue	Leaf	1	8.7	Red	2
3.1+6.4	Red	Support	2	Blue	Leaf	1	8.13	Blue	4
3.1+6.4	Red	Leaf	1	Blue	Support	3	8.17	Black	4
3.1+6.4	Red	Support	2	Blue	Support	3	8.19	Black	2
3.1+6.4	Red	Leaf	1	Blue	Leaf	1	8.8	Blue	8
3.1+6.5	Red	Leaf	1	Black	Leaf	1	8.12	Blue	6
3.1+6.5	Red	Leaf	1	Black	Support	2	8.15	Red	2
3.1+6.5	Red	Support	2	Black	Leaf	1	8.17	Black	3
3.1+6.5	Red	Support	2	Black	Support	2	8.18	Black	1
3.1+6.5	Red	Leaf	1	Black	Support	4	8.21	Black	2
3.1+6.5	Red	Support	2	Black	Support	4	8.22	Black	1
3.1+6.5	Red	Leaf	1	Black	Leaf	1	8.6	Blue	2
3.1+6.5	Red	Support	2	Black	Leaf	1	8.9	Black	1
3.1+6.6	Red	Leaf	1	Red	Leaf	1	8.14	Black	10
3.1+6.6	Red	Support	2	Red	Leaf	1	8.19	Black	5
3.1+6.6	Red	Leaf	1	Red	Support	5	8.22	Black	2
3.1+6.6	Red	Support	2	Red	Support	5	8.23	Black	1
3.1+7.1	Red	Leaf	1	Red	Leaf	1	9.1	Unknown	4
3.1+7.1	Red	Leaf	1	Red	Internal	2	9.11	Red	4
3.1+7.1	Red	Leaf	1	Red	Internal	2	9.18	Unknown	2
3.1+7.1	Red	Support	2	Red	Internal	2	9.19	Unknown	1
3.1+7.1	Red	Support	2	Red	Leaf	1	9.2	Unknown	2
3.1+7.1	Red	Support	2	Red	Internal	2	9.21	Unknown	2
3.1+7.1	Red	Leaf	1	Red	Support	2	9.4	Unknown	4
3.1+7.1	Red	Support	2	Red	Support	2	9.5	Unknown	2
3.1+7.10	Red	Leaf	1	Black	Leaf	1	9.15	Unknown	2
3.1+7.10	Red	Support	2	Black	Leaf	1	9.25	Unknown	1
3.1+7.10	Red	Leaf	1	Black	Leaf	1	9.31	Unknown	8
3.1+7.10	Red	Leaf	1	Black	Support	2	9.36	Unknown	2
3.1+7.10	Red	Support	2	Black	Leaf	1	9.39	Unknown	4
3.1+7.10	Red	Support	2	Black	Support	2	9.40	Unknown	1
3.1+7.10	Red	Leaf	1	Black	Support	5	9.45	Unknown	2
3.1+7.10	Red	Support	2	Black	Support	5	9.46	Unknown	1
3.1+7.11	Red	Leaf	1	Black	Leaf	1	9.32	Unknown	12
3.1+7.11	Red	Support	2	Black	Leaf	1	9.41	Unknown	6
3.1+7.11	Red	Leaf	1	Black	Support	6	9.46	Unknown	2

RAG Trees to be Merged	DATA FROM THE 1st TREE			DATA FROM THE 2nd TREE			RESULTS		
	Tree Color	Vert. Type	$deg(v)$	Tree Color	Vert. Type	$deg(v)$	RAG Graph	Tree Color	Total Graphs
3.1+7.11	Red	Support	2	Black	Support	6	9.47	Unknown	1
3.1+7.2	Red	Support	2	Red	Support	3	9.15	Unknown	1
3.1+7.2	Red	Leaf	1	Red	Internal	2	9.17	Unknown	2
3.1+7.2	Red	Leaf	1	Red	Leaf	1	9.2	Unknown	2
3.1+7.2	Red	Leaf	1	Red	Support	3	9.21	Unknown	2
3.1+7.2	Red	Support	2	Red	Internal	2	9.22	Unknown	1
3.1+7.2	Red	Leaf	1	Red	Internal	2	9.26	Unknown	2
3.1+7.2	Red	Support	2	Red	Leaf	1	9.3	Unknown	1
3.1+7.2	Red	Support	2	Red	Internal	2	9.44	Unknown	1
3.1+7.2	Red	Leaf	1	Red	Leaf	1	9.6	Red	4
3.1+7.2	Red	Leaf	1	Red	Support	2	9.7	Unknown	2
3.1+7.2	Red	Support	2	Red	Leaf	1	9.8	Unknown	2
3.1+7.2	Red	Support	2	Red	Support	2	9.9	Unknown	1
3.1+7.3	Red	Support	2	Red	Leaf	1	9.10	Unknown	1
3.1+7.3	Red	Leaf	1	Red	Support	2	9.12	Unknown	2
3.1+7.3	Red	Leaf	1	Red	Support	2	9.14	Unknown	2
3.1+7.3	Red	Support	2	Red	Support	2	9.16	Unknown	1
3.1+7.3	Red	Leaf	1	Red	Leaf	1	9.18	Unknown	2
3.1+7.3	Red	Support	2	Red	Support	2	9.20	Unknown	1
3.1+7.3	Red	Support	2	Red	Leaf	1	9.26	Unknown	1
3.1+7.3	Red	Leaf	1	Red	Internal	2	9.27	Red	2
3.1+7.3	Red	Leaf	1	Red	Internal	3	9.29	Unknown	2
3.1+7.3	Red	Support	2	Red	Internal	2	9.30	Unknown	1
3.1+7.3	Red	Support	2	Red	Internal	3	9.31	Unknown	1
3.1+7.3	Red	Leaf	1	Red	Leaf	1	9.4	Unknown	2
3.1+7.3	Red	Leaf	1	Red	Leaf	1	9.6	Red	2
3.1+7.3	Red	Support	2	Red	Leaf	1	9.7	Unknown	1
3.1+7.4	Red	Leaf	1	Blue	Leaf	1	9.10	Unknown	8
3.1+7.4	Red	Support	2	Blue	Leaf	1	9.13	Red	4
3.1+7.4	Red	Leaf	1	Blue	Support	3	9.22	Unknown	4
3.1+7.4	Red	Support	2	Blue	Support	3	9.25	Unknown	2
3.1+7.4	Red	Leaf	1	Blue	Internal	2	9.33	Unknown	2
3.1+7.4	Red	Support	2	Blue	Internal	2	9.35	Unknown	1
3.1+7.5	Red	Leaf	1	Blue	Support	2	9.16	Unknown	2
3.1+7.5	Red	Leaf	1	Blue	Leaf	1	9.19	Unknown	6
3.1+7.5	Red	Support	2	Blue	Support	2	9.23	Unknown	1
3.1+7.5	Red	Leaf	1	Blue	Support	4	9.31	Unknown	2
3.1+7.5	Red	Support	2	Blue	Support	4	9.32	Unknown	1
3.1+7.5	Red	Leaf	1	Blue	Internal	2	9.34	Unknown	2
3.1+7.5	Red	Support	2	Blue	Internal	2	9.37	Unknown	1
3.1+7.5	Red	Support	2	Blue	Leaf	1	9.44	Unknown	3
3.1+7.5	Red	Leaf	1	Blue	Leaf	1	9.5	Unknown	2

RAG Trees to be Merged	DATA FROM THE 1st TREE			DATA FROM THE 2nd TREE			RESULTS		
	Tree Color	Vert. Type	*deg* (v)	Tree Color	Vert. Type	*deg* (v)	RAG Graph	Tree Color	Total Graphs
3.1+7.5	Red	Support	2	Blue	Leaf	1	9.9	Unknown	1
3.1+7.6	Red	Support	2	Red	Leaf	1	9.13	Red	1
3.1+7.6	Red	Leaf	1	Red	Leaf	1	9.14	Unknown	4
3.1+7.6	Red	Leaf	1	Red	Support	2	9.24	Unknown	2
3.1+7.6	Red	Support	2	Red	Leaf	1	9.24	Unknown	2
3.1+7.6	Red	Leaf	1	Red	Leaf	1	9.26	Unknown	2
3.1+7.6	Red	Support	2	Red	Support	2	9.28	Unknown	1
3.1+7.6	Red	Leaf	1	Red	Support	3	9.30	Unknown	2
3.1+7.6	Red	Support	2	Red	Leaf	1	9.33	Unknown	1
3.1+7.6	Red	Support	2	Red	Support	3	9.36	Unknown	1
3.1+7.6	Red	Leaf	1	Red	Internal	3	9.38	Unknown	2
3.1+7.6	Red	Support	2	Red	Internal	3	9.39	Unknown	1
3.1+7.6	Red	Leaf	1	Red	Leaf	1	9.8	Unknown	2
3.1+7.7	Red	Leaf	1	Blue	Leaf	1	9.21	Unknown	4
3.1+7.7	Red	Support	2	Blue	Leaf	1	9.22	Unknown	2
3.1+7.7	Red	Leaf	1	Blue	Leaf	1	9.29	Unknown	4
3.1+7.7	Red	Leaf	1	Blue	Support	2	9.30	Unknown	4
3.1+7.7	Red	Support	2	Blue	Support	2	9.37	Unknown	2
3.1+7.7	Red	Support	2	Blue	Leaf	1	9.38	Unknown	2
3.1+7.7	Red	Leaf	1	Blue	Internal	4	9.43	Unknown	2
3.1+7.7	Red	Support	2	Blue	Internal	3	9.45	Unknown	1
3.1+7.8	Red	Leaf	1	Blue	Leaf	1	9.11	Red	6
3.1+7.8	Red	Support	2	Blue	Leaf	1	9.17	Unknown	3
3.1+7.8	Red	Leaf	1	Blue	Support	2	9.27	Red	6
3.1+7.8	Red	Support	2	Blue	Support	2	9.34	Unknown	3
3.1+7.8	Red	Leaf	1	Blue	Internal	3	9.42	Unknown	2
3.1+7.8	Red	Support	2	Blue	Internal	3	9.43	Unknown	1
3.1+7.9	Red	Leaf	1	Black	Leaf	1	9.20	Unknown	4
3.1+7.9	Red	Support	2	Black	Leaf	1	9.28	Unknown	2
3.1+7.9	Red	Support	2	Black	Leaf	1	9.35	Unknown	3
3.1+7.9	Red	Leaf	1	Black	Support	3	9.37	Unknown	2
3.1+7.9	Red	Leaf	1	Black	Support	4	9.39	Unknown	2
3.1+7.9	Red	Support	2	Black	Support	3	9.40	Unknown	1
3.1+7.9	Red	Support	2	Black	Support	3	9.41	Unknown	1
3.1+7.9	Red	Leaf	1	Black	Leaf	1	9.44	Unknown	6
4.1+4.1	Red	Leaf	1	Red	Leaf	1	7.1	Red	4
4.1+4.1	Red	Leaf	1	Red	Support	2	7.3	Red	8
4.1+4.1	Red	Support	2	Red	Support	2	7.7	Blue	4
4.1+4.2	Red	Support	2	Blue	Support	3	7.10	Black	2
4.1+4.2	Red	Leaf	1	Blue	Leaf	1	7.2	Red	6
4.1+4.2	Red	Leaf	1	Blue	Support	3	7.5	Blue	2

RAG Trees to be Merged	DATA FROM THE 1st TREE			DATA FROM THE 2nd TREE			RESULTS		
	Tree Color	Vert. Type	*deg* (v)	Tree Color	Vert. Type	*deg* (v)	RAG Graph	Tree Color	Total Graphs
4.1+4.2	Red	Support	2	Blue	Leaf	1	7.6	Red	6
4.1+5.1	Red	Leaf	1	Red	Leaf	1	8.1	Blue	4
4.1+5.1	Red	Leaf	1	Red	Internal	2	8.10	Red	2
4.1+5.1	Red	Support	2	Red	Support	2	8.12	Blue	4
4.1+5.1	Red	Support	2	Red	Internal	2	8.20	Red	2
4.1+5.1	Red	Support	2	Red	Leaf	1	8.3	Red	4
4.1+5.1	Red	Leaf	1	Red	Support	2	8.5	Red	4
4.1+5.2	Red	Support	2	Red	Support	3	8.12	Blue	2
4.1+5.2	Red	Support	2	Red	Support	2	8.17	Black	2
4.1+5.2	Red	Leaf	1	Red	Leaf	1	8.2	Blue	2
4.1+5.2	Red	Support	2	Red	Support	3	8.21	Black	2
4.1+5.2	Red	Leaf	1	Red	Leaf	1	8.3	Red	4
4.1+5.2	Red	Support	2	Red	Leaf	1	8.7	Red	2
4.1+5.2	Red	Leaf	1	Red	Support	2	8.8	Blue	2
4.1+5.2	Red	Support	2	Red	Leaf	1	8. 11	Red	4
4.1+5.3	Red	Leaf	1	Red	Support	4	8.14	Black	2
4.1+5.3	Red	Support	2	Red	Leaf	1	8.15	Red	8
4.1+5.3	Red	Support	2	Red	Support	4	8.22	Black	2
4.1+5.3	Red	Leaf	1	Red	Leaf	1	8.6	Blue	8
4.1+6.1	Red	Leaf	1	Blue	Leaf	1	9.1	Unknown	4
4.1+6.1	Red	Leaf	1	Blue	Internal	2	9.18	Unknown	4
4.1+6.1	Red	Support	2	Blue	Support	2	9.21	Unknown	4
4.1+6.1	Red	Support	2	Blue	Internal	2	9.29	Unknown	4
4.1+6.1	Red	Support	2	Blue	Leaf	1	9.4	Unknown	4
4.1+6.1	Red	Leaf	1	Blue	Support	2	9.6	Red	4
4.1+6.2	Red	Leaf	1	Red	Support	2	9.10	Unknown	2
4.1+6.2	Red	Support	2	Red	Leaf	1	9.14	Unknown	4
4.1+6.2	Red	Leaf	1	Red	Support	3	9.19	Unknown	2
4.1+6.2	Red	Leaf	1	Red	Leaf	1	9.2	Unknown	2
4.1+6.2	Red	Support	2	Red	Support	2	9.22	Unknown	2
4.1+6.2	Red	Leaf	1	Red	Internal	2	9.26	Unknown	2
4.1+6.2	Red	Support	2	Red	Support	3	9.31	Unknown	2
4.1+6.2	Red	Support	2	Red	Internal	2	9.38	Unknown	2
4.1+6.2	Red	Leaf	1	Red	Leaf	1	9.6	Red	4
4.1+6.2	Red	Support	2	Red	Leaf	1	9.7	Unknown	2
4.1+6.3	Red	Leaf	1	Blue	Leaf	1	9.11	Red	2
4.1+6.3	Red	Support	2	Blue	Leaf	1	9.12	Unknown	4
4.1+6.3	Red	Leaf	1	Blue	Support	2	9.14	Unknown	4
4.1+6.3	Red	Support	2	Blue	Leaf	1	9.27	Red	2
4.1+6.3	Red	Leaf	1	Blue	Support	3	9.29	Unknown	2
4.1+6.3	Red	Support	2	Blue	Support	2	9.30	Unknown	4

RAG Trees to be Merged	DATA FROM THE 1st TREE			DATA FROM THE 2nd TREE			RESULTS		
	Tree Color	Vert. Type	*deg* (v)	Tree Color	Vert. Type	*deg* (v)	RAG Graph	Tree Color	Total Graphs
4.1+6.3	Red	Leaf	1	Blue	Leaf	1	9.4	Unknown	4
4.1+6.3	Red	Support	2	Blue	Support	3	9.43	Unknown	2
4.1+6.4	Red	Support	2	Blue	Leaf	1	9.24	Unknown	8
4.1+6.4	Red	Support	2	Blue	Support	3	9.39	Unknown	4
4.1+6.4	Red	Leaf	1	Blue	Support	3	9.44	Unknown	4
4.1+6.4	Red	Leaf	1	Blue	Leaf	1	9.8	Unknown	8
4.1+6.5	Red	Support	2	Black	Leaf	1	9.16	Unknown	2
4.1+6.5	Red	Leaf	1	Black	Support	2	9.20	Unknown	2
4.1+6.5	Red	Leaf	1	Black	Leaf	1	9.21	Unknown	6
4.1+6.5	Red	Support	2	Black	Leaf	1	9.30	Unknown	6
4.1+6.5	Red	Leaf	1	Black	Support	4	9.31	Unknown	2
4.1+6.5	Red	Support	2	Black	Support	2	9.37	Unknown	2
4.1+6.5	Red	Support	2	Black	Support	4	9.45	Unknown	2
4.1+6.5	Red	Leaf	1	Black	Leaf	1	9.5	Unknown	2
4.1+6.6	Red	Leaf	1	Red	Leaf	1	9.15	Unknown	10
4.1+6.6	Red	Leaf	1	Red	Support	5	9.32	Unknown	2
4.1+6.6	Red	Support	2	Red	Leaf	1	9.36	Unknown	10
4.1+6.6	Red	Support	2	Red	Support	5	9.46	Unknown	2
4.2+4.2	Blue	Support	3	Blue	Support	3	7.11	Black	1
4.2+4.2	Blue	Leaf	1	Blue	Leaf	1	7.4	Blue	9
4.2+4.2	Blue	Leaf	1	Blue	Support	3	7.9	Black	6
4.2+5.1	Blue	Support	3	Red	Support	2	8.14	Black	2
4.2+5.1	Blue	Leaf	1	Red	Internal	2	8.16	Blue	3
4.2+5.1	Blue	Leaf	1	Red	Leaf	1	8.2	Blue	6
4.2+5.1	Blue	Support	3	Red	Internal	2	8.21	Black	1
4.2+5.1	Blue	Support	3	Red	Leaf	1	8.6	Blue	2
4.2+5.1	Blue	Leaf	1	Red	Support	2	8.8	Blue	6
4.2+5.2	Blue	Leaf	1	Red	Support	2	8.13	Blue	3
4.2+5.2	Blue	Support	3	Red	Leaf	1	8.15	Red	2
4.2+5.2	Blue	Leaf	1	Red	Support	3	8.17	Black	3
4.2+5.2	Blue	Support	3	Red	Support	2	8.19	Black	1
4.2+5.2	Blue	Support	3	Red	Support	3	8.22	Black	1
4.2+5.2	Blue	Leaf	1	Red	Leaf	1	8.4	Blue	3
4.2+5.2	Blue	Leaf	1	Red	Leaf	1	8.7	Red	6
4.2+5.2	Blue	Support	3	Red	Leaf	1	8.9	Black	1
4.2+5.3	Blue	Support	3	Red	Leaf	1	8.18	Black	4
4.2+5.3	Blue	Leaf	1	Red	Support	4	8.19	Black	3
4.2+5.3	Blue	Support	3	Red	Support	4	8.23	Black	1
4.2+5.3	Blue	Leaf	1	Red	Leaf	1	8.9	Black	12
4.2+6.1	Blue	Support	3	Blue	Support	2	9.15	Unknown	2
4.2+6.1	Blue	Leaf	1	Blue	Leaf	1	9.2	Unknown	6
4.2+6.1	Blue	Leaf	1	Blue	Internal	2	9.26	Unknown	6

RAG Trees to be Merged	DATA FROM THE 1st TREE			DATA FROM THE 2nd TREE			RESULTS		
	Tree Color	Vert. Type	*deg* (v)	Tree Color	Vert. Type	*deg* (v)	RAG Graph	Tree Color	Total Graphs
4.2+6.1	Blue	Support	3	Blue	Internal	2	9.31	Unknown	2
4.2+6.1	Blue	Support	3	Blue	Leaf	1	9.5	Unknown	2
4.2+6.1	Blue	Leaf	1	Blue	Support	2	9.8	Unknown	6
4.2+6.2	Blue	Leaf	1	Red	Leaf	1	9.10	Unknown	6
4.2+6.2	Blue	Leaf	1	Red	Support	2	9.13	Red	3
4.2+6.2	Blue	Support	3	Red	Leaf	1	9.20	Unknown	2
4.2+6.2	Blue	Support	3	Red	Support	2	9.25	Unknown	1
4.2+6.2	Blue	Leaf	1	Red	Leaf	1	9.3	Unknown	3
4.2+6.2	Blue	Support	3	Red	Support	3	9.32	Unknown	1
4.2+6.2	Blue	Leaf	1	Red	Internal	2	9.33	Unknown	3
4.2+6.2	Blue	Support	3	Red	Internal	2	9.39	Unknown	1
4.2+6.2	Blue	Leaf	1	Red	Support	3	9.44	Unknown	3
4.2+6.2	Blue	Support	3	Red	Leaf	1	9.9	Unknown	1
4.2+6.3	Blue	Support	3	Blue	Leaf	1	9.16	Unknown	2
4.2+6.3	Blue	Leaf	1	Blue	Leaf	1	9.17	Unknown	3
4.2+6.3	Blue	Leaf	1	Blue	Support	2	9.24	Unknown	6
4.2+6.3	Blue	Support	3	Blue	Leaf	1	9.34	Unknown	1
4.2+6.3	Blue	Support	3	Blue	Support	2	9.36	Unknown	2
4.2+6.3	Blue	Leaf	1	Blue	Internal	3	9.38	Unknown	3
4.2+6.3	Blue	Support	3	Blue	Internal	3	9.45	Unknown	1
4.2+6.3	Blue	Leaf	1	Blue	Leaf	1	9.7	Unknown	6
4.2+6.4	Blue	Leaf	1	Blue	Leaf	1	9.13	Red	12
4.2+6.4	Blue	Support	3	Blue	Leaf	1	9.28	Unknown	4
4.2+6.4	Blue	Leaf	1	Blue	Support	3	9.35	Unknown	6
4.2+6.4	Blue	Support	3	Blue	Support	3	9.41	Unknown	2
4.2+6.5	Blue	leaf	1	Black	Leaf	1	9.22	Unknown	9
4.2+6.5	Blue	Support	3	Black	Leaf	1	9.23	Unknown	1
4.2+6.5	Blue	leaf	1	Black	Support	2	9.28	Unknown	3
4.2+6.5	Blue	Support	3	Black	Leaf	1	9.37	Unknown	3
4.2+6.5	Blue	Leaf	1	Black	Support	4	9.39	Unknown	3
4.2+6.5	Blue	Support	3	Black	Support	2	9.40	Unknown	1
4.2+6.5	Blue	Support	3	Black	Support	4	9.46	Unknown	1
4.2+6.5	Blue	leaf	1	Black	Leaf	1	9.9	Unknown	3
4.2+6.6	Blue	Leaf	1	Red	Leaf	1	9.25	Unknown	15
4.2+6.6	Blue	Support	3	Red	Leaf	1	9.40	Unknown	5
4.2+6.6	Blue	Leaf	1	Red	Support	5	9.41	Unknown	3
4.2+6.6	Blue	Support	3	Red	Support	5	9.47	Unknown	1
5.1+5.1	Red	leaf	1	Red	Leaf	1	9.1	Unknown	4
5.1+5.1	Red	Leaf	1	Red	Support	2	9.6	Red	8
5.1+5.1	Red	Support	2	Red	Support	2	9.19	Unknown	4

RAG Trees to be Merged	DATA FROM THE 1st TREE			DATA FROM THE 2nd TREE			RESULTS		
	Tree Color	Vert. Type	*deg* (v)	Tree Color	Vert. Type	*deg* (v)	RAG Graph	Tree Color	Total Graphs
5.1+5.1	Red	leaf	1	Red	Internal	2	9.11	Red	4
5.1+5.1	Red	Leaf	1	Red	Internal	2	9.29	Unknown	4
5.1+5.1	Red	Internal	2	Red	Internal	2	9.42	Unknown	1
5.1+5.2	Red	Support	2	Red	Leaf	1	9.10	Unknown	2
5.1+5.2	Red	Support	2	Red	Leaf	1	9.14	Unknown	4
5.1+5.2	Red	Internal	2	Red	Leaf	1	9.17	Unknown	1
5.1+5.2	Red	Leaf	1	Red	Leaf	1	9.2	Unknown	2
5.1+5.2	Red	Leaf	1	Red	Support	3	9.21	Unknown	2
5.1+5.2	Red	Internal	2	Red	Leaf	1	9.27	Red	2
5.1+5.2	Red	Support	2	Red	Support	3	9.31	Unknown	2
5.1+5.2	Red	Internal	2	Red	Support	2	9.38	Unknown	1
5.1+5.2	Red	Leaf	1	Red	Leaf	1	9.4	Unknown	4
5.1+5.2	Red	Internal	2	Red	Support	3	9.43	Unknown	1
5.1+5.2	Red	Support	2	Red	Support	2	9.44	Unknown	2
5.1+5.2	Red	Leaf	1	Red	Support	2	9.8	Unknown	2
5.1+5.3	Red	Leaf	1	Red	Support	4	9.15	Unknown	2
5.1+5.3	Red	Support	2	Red	Leaf	1	9.20	Unknown	8
5.1+5.3	Red	Support	2	Red	Support	4	9.32	Unknown	2
5.1+5.3	Red	Internal	2	Red	Leaf	1	9.34	Unknown	4
5.1+5.3	Red	Internal	2	Red	Support	4	9.45	Unknown	1
5.1+5.3	Red	Leaf	1	Red	Leaf	1	9.5	Unknown	8
5.2+5.2	Red	Leaf	1	Red	Leaf	1	9.12	Unknown	4
5.2+5.2	Red	Support	2	Red	Leaf	1	9.13	Red	2
5.2+5.2	Red	Support	3	Red	Leaf	1	9.22	Unknown	2
5.2+5.2	Red	Leaf	1	Red	Support	2	9.24	Unknown	4
5.2+5.2	Red	Leaf	1	Red	Leaf	1	9.3	Unknown	1
5.2+5.2	Red	Leaf	1	Red	Support	3	9.30	Unknown	4
5.2+5.2	Red	Support	2	Red	Support	2	9.35	Unknown	1
5.2+5.2	Red	Support	3	Red	Support	2	9.39	Unknown	2
5.2+5.2	Red	Support	3	Red	Support	3	9.45	Unknown	1
5.2+5.2	Red	Leaf	1	Red	Leaf	1	9.7	Unknown	4
5.2+5.3	Red	Leaf	1	Red	Leaf	1	9.16	Unknown	8
5.2+5.3	Red	Leaf	1	Red	Support	4	9.25	Unknown	1
5.2+5.3	Red	Support	2	Red	Leaf	1	9.28	Unknown	4
5.2+5.3	Red	Leaf	1	Red	Support	4	9.36	Unknown	2
5.2+5.3	Red	Support	3	Red	Leaf	1	9.37	Unknown	4
5.2+5.3	Red	Support	2	Red	Support	4	9.41	Unknown	1
5.2+5.3	Red	Support	3	Red	Support	4	9.46	Unknown	1
5.2+5.3	Red	Leaf	1	Red	Leaf	1	9.9	Unknown	4
5.3+5.3	Red	Leaf	1	Red	Leaf	1	9.23	Unknown	16
5.3+5.3	Red	Support	4	Red	Leaf	1		Unknown	8
5.3+5.3	Red	Support	4	Red	Support	4	9.47	Unknown	1
